# Household Economic Burden and Healthcare Utilization Among Adults With Type 2 Diabetes Mellitus: A Hospital-Based Cost-of-Illness Study in Rural North India

**DOI:** 10.7759/cureus.114390

**Published:** 2026-08-12

**Authors:** Manish Kundu, Shalini Ray, Manisha Singh, Saranya K

**Affiliations:** 1 Community Medicine, Maharaja Agrasen Medical College, Agroha, Hisar, IND; 2 Community Medicine, PESU Institute of Medical Sciences and Research, Bengaluru, IND

**Keywords:** cost of illness, economic burden of healthcare, financial health protection, health economics, type 2 diabetes mellitus

## Abstract

Background

Type 2 diabetes mellitus (T2DM) is a major public health challenge that places a substantial economic burden on households because of lifelong treatment and monitoring requirements. Evidence on the household financial burden of diabetes in rural India remains limited. This study estimated the household cost of illness associated with T2DM and assessed healthcare utilization among adults attending a rural tertiary care teaching hospital in North India.

Methods

A hospital-based cross-sectional study was conducted among 400 adults with T2DM who had been receiving treatment for at least six months. Participants were enrolled consecutively. Data were collected through face-to-face interviews using a pretested semi-structured questionnaire to capture sociodemographic characteristics, clinical profile, healthcare utilization, and diabetes-related expenditure. Household costs were estimated from the patient perspective and categorized into direct medical, direct nonmedical, and indirect costs.

Results

The mean age of participants was 55.2 ± 8.5 years, and 58.3% had diabetes for 5-10 years. The mean annual household expenditure attributable to diabetes was ₹15,204 (₹1,267/month), with direct medical costs accounting for 76.7% of total expenditure. Most households (73.5%) incurred annual diabetes-related expenses between ₹10,001 and ₹20,000. Most participants (85.5%) used outpatient care, and 96.5% preferred allopathic treatment. Overall, 14.5% of households spent more than 30% of their monthly household income on diabetes care, indicating substantial financial vulnerability.

Conclusions

T2DM imposes a considerable economic burden on rural households, with direct medical expenditure being the principal cost driver. Strengthening primary healthcare, improving access to affordable medicines, and expanding financial risk protection mechanisms are essential to reducing out-of-pocket expenditures.

## Introduction

Type 2 diabetes mellitus (T2DM) presents a significant and escalating challenge for health systems worldwide. Despite improvements in diabetes management that have enhanced survival rates, the prevalence of diabetes continues to increase, especially in developing countries. India carries one of the largest global burdens of diabetes [1]. According to the International Diabetes Federation, nearly 89.8 million adults in India were living with diabetes in 2024, positioning India as the “diabetes capital” [2]. The National Family Health Survey (NFHS-6) reported a high prevalence of elevated blood sugar among 20.9% of men and 17.8% of women [3]. Beyond its clinical implications, diabetes is increasingly recognized as a major socioeconomic issue due to the need for lifelong treatment, frequent monitoring, and ongoing management of chronic complications, all of which contribute to substantial healthcare expenditures for individuals and health systems [2-4].

Such health expenditures gradually deplete household financial resources, especially in contexts where healthcare financing is primarily based on out-of-pocket payments. As a result, patients may postpone consultations, omit medications, reduce monitoring frequency, or borrow funds to cover treatment expenses, negatively impacting both health outcomes and household economic stability [5-7]. The economic burden of diabetes is typically assessed using the cost-of-illness (COI) framework, which classifies expenditures into direct medical costs, direct nonmedical costs, and indirect costs. Direct medical costs encompass spending on medications, physician consultations, laboratory investigations, and hospital admissions. Direct nonmedical costs include transportation, accommodation, and food expenses incurred during healthcare-seeking. Indirect costs result from productivity losses due to absenteeism, disability, premature retirement, and caregiver involvement [8]. Despite the expansion of national programs targeting noncommunicable diseases and the introduction of publicly financed health insurance schemes, a substantial proportion of patients continue to shoulder significant treatment costs, particularly in rural settings.

While many studies have investigated the epidemiology and clinical characteristics of diabetes in India, few have comprehensively assessed both health-seeking behavior and the household economic burden of diabetes using a COI approach. Analyzing healthcare utilization patterns alongside the economic impact of long-term diabetes management is crucial for informing interventions that enhance affordability, reinforce economic risk protection, and advance progress toward Universal Health Coverage. In this context, the present study aimed to (i) estimate the direct, indirect, and total economic burden associated with T2DM and (ii) characterize the health-seeking behavior of adults attending a rural tertiary care teaching hospital in Haryana, North India.

## Materials and methods

A hospital-based cross-sectional COI study was conducted among adults with T2DM attending outpatient and inpatient services in the Department of Medicine at a rural tertiary care teaching hospital in Haryana, North India. The hospital primarily serves rural and semi-urban populations from Haryana and neighboring states. Adults aged 18 years and above with a confirmed diagnosis of T2DM who had been receiving treatment for at least six months and attended the medicine outpatient department or were admitted to the inpatient department during the study period were eligible for inclusion. Patients with type 1 diabetes mellitus, gestational diabetes, those who were critically ill and unable to participate in the interview, and individuals unwilling to provide written informed consent were excluded.

A total of 400 participants were enrolled using consecutive sampling. As the primary objective was to estimate the household economic burden of diabetes rather than test a specific hypothesis, all eligible patients presenting during the study period were invited to participate. Universal sampling was used, and of the 429 patients assessed for eligibility, 400 fulfilled the inclusion criteria and consented to participate. Recruiting all eligible participants during the study period ensured adequate representation of the hospital population and provided sufficient precision for estimating household expenditure and healthcare utilization patterns.

Data collection involved face-to-face interviews using a predesigned and pretested semi-structured questionnaire (Appendix A). Prior to the main study, the questionnaire was pilot-tested among 40 adults with T2DM at the Rural Health Training Centre. Minor modifications were made based on the pilot findings to enhance clarity, sequencing, and comprehensiveness. The questionnaire consisted of four sections: (i) sociodemographic characteristics; (ii) healthcare-seeking behavior; (iii) clinical profile, including duration of diabetes, treatment, comorbidities, and complications; and (iv) diabetes-related household expenditure. Socioeconomic status was assessed using the Modified B.G. Prasad Scale applicable during the study period [9].

The household economic burden of diabetes was assessed using the COI approach from the household perspective. Participants reported diabetes-related expenditures incurred during the reference period, which were then annualized to estimate annual household expenditures. Direct medical costs included expenditure on physician consultations, antidiabetic medications, insulin, insulin delivery devices, laboratory investigations, home blood glucose monitoring supplies, and diabetes-related hospitalizations. Consultation costs were estimated from the number of healthcare visits and the consultation fees paid, while medication costs were calculated from the quantities of medicines and consumables purchased and their prevailing market prices. Expenses incurred for laboratory investigations performed outside the study hospital and, where applicable, for the purchase of a glucometer were also included.

Direct nonmedical costs included transportation, food, accommodation, and additional dietary expenses incurred while seeking diabetes care in the past one year. Indirect costs were estimated using the human capital approach and encompassed productivity losses due to patient and caregiver work absenteeism. Income loss was determined by multiplying the number of workdays lost because of diabetes-related illness or healthcare visits by the participant’s average daily income. Total annual household expenditure attributable to diabetes was calculated as the sum of direct medical, direct nonmedical, and indirect costs. Households were considered to have a high financial burden when diabetes-related expenditure exceeded 30% of their monthly household income [10].

Data were entered into Microsoft Excel (Microsoft Corporation, Redmond, WA, USA) and analyzed using IBM SPSS Statistics for Windows, version 26.0 (released 2018; IBM Corp., Armonk, NY, USA). Continuous variables are presented as mean ± SD. Comparisons between two groups were conducted using the independent Student’s t-test, while comparisons among three or more groups utilized one-way ANOVA. A two-sided p-value <0.05 was considered statistically significant. A Sankey diagram was constructed to visually represent the flow from healthcare utilization pathways to household financial burden, illustrating the relationships among service utilization, treatment preferences, expenditure components, and the proportion of household income allocated to diabetes care.

The study was approved by the Institutional Ethics Committee before commencement of data collection. Written informed consent was obtained from all participants before enrollment. Participant confidentiality was maintained throughout the study by anonymizing the dataset, and the study was conducted in accordance with the principles of the Declaration of Helsinki.

## Results

A total of 400 adults with T2DM participated in the study. The mean age was 55.20 ± 8.54 years. Nearly half of the participants were in the 45- to 60-year age group, and 159 (39.8%) were older than 60 years. Males comprised 202 (50.5%) of the study population. The majority resided in rural areas, reflecting the hospital’s catchment population. Approximately half belonged to the middle socioeconomic class. Most participants, 233 (58.3%), had diabetes for 5-10 years, 127 (31.8%) for less than five years, and 40 (10%) for more than 10 years (Table 1).

**Table 1:** Sociodemographic profile of the study participants (N = 400).

Characteristic	n	%
Age group (years)		
18-44	43	10.8
45-60	198	49.5
>60	159	39.8
Sex		
Male	202	50.5
Female	198	49.5
Residence		
Rural	286	71.5
Urban	114	28.5
Duration of diabetes		
<5 years	127	31.8
5-10 years	233	58.3
>10 years	40	10
Socioeconomic status		
Upper/upper middle	86	21.5
Middle	201	50.3
Lower middle/lower	113	28.2

Household cost of diabetes

Table 2 indicates that the mean annual household expenditure attributable to diabetes was ₹15,204, corresponding to a mean monthly expenditure of ₹1,267. Direct costs (medical and nonmedical) constituted the largest share, averaging ₹972 per month, while indirect costs, mainly from productivity and wage losses, averaged ₹295 per month. Consequently, direct expenditures represented approximately 76.7% of total monthly diabetes-related costs, with indirect costs accounting for 23.3%.

**Table 2:** Household expenditure attributable to diabetes.

Cost component	Mean cost (₹)	Contribution (%)
Direct cost/month	972	76.7
Indirect cost/month	295	23.3
Total monthly expenditure	1,267	100
Total annual expenditure	15,204	-

Distribution of annual expenditure

Table 3 demonstrates that annual diabetes-related expenditure varied among households. Most participants, 294 (73.5%), reported annual expenditures between ₹10,001 and ₹20,000, while 96 (24%) spent less than ₹10,000 annually. Only 10 (2.5%) reported annual expenditures exceeding ₹20,000. These results suggest that diabetes imposes a persistent financial obligation on most households, even within a predominantly rural population.

**Table 3:** Distribution of annual diabetes-related expenditure (N = 400).

Annual expenditure (₹)	n	%
<10,000	96	24
10,001-20,000	294	73.5
>20,000	10	2.5

**Figure 1:**
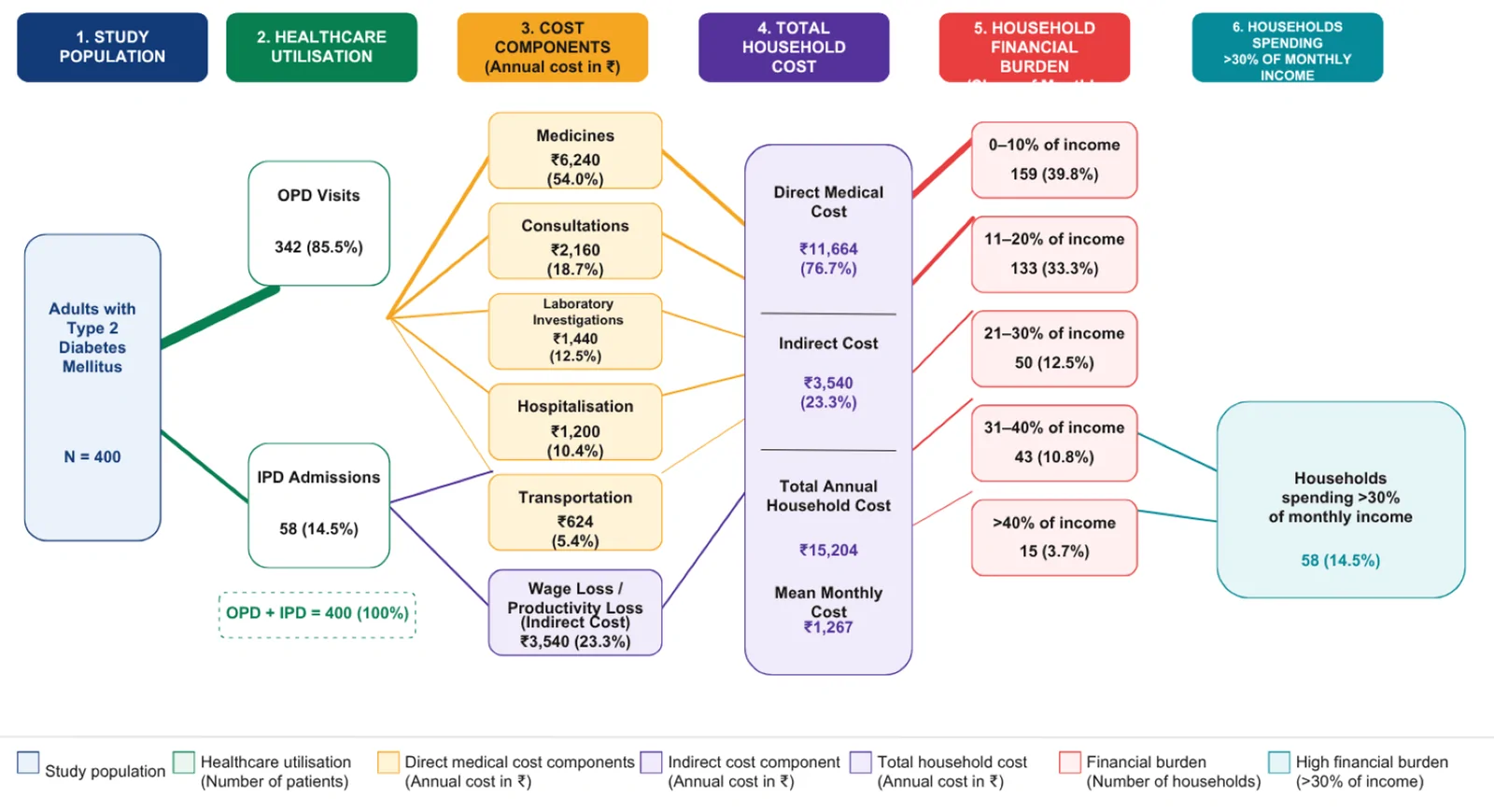
Sankey diagram showing the pathway from healthcare utilization to household financial burden. Percentages may not add up to 100% due to rounding.

Figure 1 illustrates the pathway connecting healthcare utilization to the economic burden experienced by adults with T2DM. The diagram starts with the 400 study participants, depicting their healthcare utilization patterns and the subsequent allocation into various cost components that collectively contribute to the household’s overall financial burden.

The majority of participants, 342 (85.5%), utilized outpatient services for diabetes management, whereas 58 (14.5%) required inpatient care during the study period. Nearly all participants, 386 (96.5%), preferred allopathic treatment, indicating that conventional medical care was the predominant mode of diabetes management in the study population.

The final section of the Sankey diagram demonstrates the impact of these expenditures on household finances. Approximately 39.8% of participants spent up to 10% of their monthly household income on diabetes care, while 33.3% spent 11-20%. A further 12.5% allocated 21-30% of their monthly income to diabetes management. Importantly, 14.5% of households spent more than 30% of their monthly household income on diabetes-related healthcare, highlighting a substantial financial burden among a considerable proportion of the study population.

Overall, diabetes imposed a significant financial burden on households in this rural population. Direct medical expenditure represented more than three-quarters of total diabetes-related costs, indicating that medicines, consultations, and investigations were the primary drivers of household spending. Although most participants incurred annual expenditures between ₹10,001 and ₹20,000, nearly one in seven households allocated more than 30% of their monthly income to diabetes care. These findings indicate that, despite the availability of healthcare services, long-term diabetes management continues to expose socioeconomically disadvantaged households to considerable financial vulnerability.

Factors associated with household expenditure

Mean annual household expenditure was significantly higher among participants with longer duration of diabetes, insulin use, diabetes-related complications, and higher socioeconomic status (Table 4). Expenditure was highest among those with diabetes for >10 years (₹20,265 ± 4,902, p < 0.001). Participants receiving insulin had higher expenditure than those receiving oral drugs alone (₹17,912 ± 4,208 vs. ₹13,785 ± 2,954, p < 0.001). Similarly, those with complications had higher expenditure than those without complications (₹17,498 ± 4,364 vs. ₹13,694 ± 2,881, p < 0.001). Urban participants also had higher expenditure than rural participants (₹16,045 ± 4,215 vs. ₹14,870 ± 3,620, p = 0.005). Expenditure also differed significantly across socioeconomic groups (p < 0.001).

**Table 4:** Comparison of mean annual household expenditure attributable to T2DM according to participant characteristics (N = 400). Data are presented as mean ± SD. Comparisons between two independent groups were performed using the independent Student’s t-test, while comparisons among three groups were performed using one-way ANOVA. A two-tailed p-value <0.05 was considered statistically significant. T2DM, type 2 diabetes mellitus

Variable	Groups	n	Mean ± SD	Test statistic	p-value
Residence	Rural	286	14,870 ± 3,620	t(398) = 2.79	0.005
	Urban	114	16,045 ± 4,215		
Duration of diabetes	<5 years	127	12,940 ± 2,845	F(2,397) = 66.72	<0.001
	5-10 years	233	15,385 ± 3,618		
	>10 years	40	20,265 ± 4,902		
Treatment modality	Oral drugs	262	13,785 ± 2,954	t(398) = 11.41	<0.001
	Insulin ± oral	138	17,912 ± 4,208		
Diabetes complications	Absent	241	13,694 ± 2,881	t(398) = 10.50	<0.001
	Present	159	17,498 ± 4,364		
Socioeconomic status	Upper/upper middle	86	16,732 ± 4,116	F(2,397) = 10.86	<0.001
	Middle	201	15,126 ± 3,485		
	Lower middle/lower	113	14,298 ± 3,671		

Factors associated with high household financial burden

A high household financial burden, defined as expenditures exceeding 30% of monthly household income on diabetes care, was observed in 58 (14.5%) participants. The proportion of households experiencing a high financial burden increased significantly with longer duration of diabetes, insulin-based treatment, and the presence of diabetes-related complications (p < 0.001). Participants with diabetes for more than 10 years had the highest prevalence of financial burden (40.0%), followed by those receiving insulin therapy, 38 (27.5%), and those with diabetes-related complications, 40 (25.2%). Financial burden also varied significantly across socioeconomic groups, with the highest proportion among lower socioeconomic households (21.2%; p = 0.013). Although rural participants reported a higher prevalence of financial burden than urban participants (16.1% vs. 10.5%), this difference was not statistically significant (p = 0.144) (Table 5).

**Table 5:** Association between participant characteristics and high household financial burden (>30% of monthly household income spent on diabetes care) (N = 400).

Variable	Total (n)	High financial burden, n (%)	No high financial burden, n (%)	χ² value	p-value
Residence					
Rural	286	46 (16.1)	240 (83.9)	2.13	0.144
Urban	114	12 (10.5)	102 (89.5)		
Duration of diabetes					
<5 years	127	8 (6.3)	119 (93.7)	21.46	<0.001
5-10 years	233	34 (14.6)	199 (85.4)		
>10 years	40	16 (40.0)	24 (60.0)		
Treatment modality					
Oral hypoglycemic agents only	262	20 (7.6)	242 (92.4)	31.82	<0.001
Insulin ± oral drugs	138	38 (27.5)	100 (72.5)		
Diabetes-related complications					
Absent	241	18 (7.5)	223 (92.5)	29.63	<0.001
Present	159	40 (25.2)	119 (74.8)		
Socioeconomic status					
Upper/upper middle	86	7 (8.1)	79 (91.9)	8.74	0.013
Middle	201	27 (13.4)	174 (86.6)		
Lower middle/lower	113	24 (21.2)	89 (78.8)		

## Discussion

The present study found that the mean annual household expenditure attributable to T2DM was ₹15,204, with direct medical costs comprising the largest share of total expenditure. This result aligns with recent evidence from North India. For example, a community-based COI study in urban Haryana by Bansal et al. reported a mean annual cost of ₹17,113, with direct medical expenditure accounting for 54.7% of the total cost and medicines representing the largest proportion of household spending [11]. Differences in study settings may account for the slightly lower annual expenditure observed in the present study, as participants were recruited from a rural tertiary care hospital offering subsidized investigations and medicines, whereas Bansal et al. evaluated urban households from a community perspective [11]. Nonetheless, both studies consistently demonstrate that long-term diabetes management imposes a considerable financial burden.

The predominance of direct medical expenditure observed in this study is consistent with findings from western India. In a longitudinal COI study, Prajapati et al. reported a mean annual expenditure of ₹12,392, with approximately 74% attributed to direct medical costs and 24% to indirect costs [12]. As in the present study, medicines accounted for the largest share of direct expenditure, highlighting that pharmacological management remains the primary driver of diabetes-related household costs. The marginally higher expenditure observed in this study may be due to inflation, rising medication prices, and longer diabetes duration among participants.

Higher annual household expenditure among participants with longer diabetes duration, insulin therapy, and diabetes-related complications aligns with previous studies from India and Bangladesh. Bansal et al. identified disease severity and treatment intensity as major contributors to household expenditure, while Prajapati et al. found significantly higher costs among insulin-treated patients and those with long-standing diabetes [11,12]. Similarly, Afroz et al. reported that direct medical expenditure increased substantially among patients requiring insulin and those with diabetes-related complications, reflecting additional costs for medications, glucose monitoring, and chronic complication management [13].

Notably, 14.5% of households in the present study spent more than 30% of their monthly household income on diabetes care, indicating substantial financial vulnerability among socioeconomically disadvantaged families. Although catastrophic health expenditure was not formally calculated using World Health Organization thresholds, this proportion suggests a considerable risk of financial hardship. Recent research by Bansal et al. found that 10.1% of urban households experienced catastrophic health expenditure due to diabetes, and nearly one-fifth experienced impoverishment after covering diabetes-related healthcare costs [11]. The slightly higher proportion observed in this rural population may reflect lower household incomes, greater reliance on out-of-pocket expenditure, and reduced economic security among rural families.

Similarly, Engelgau et al. demonstrated that noncommunicable diseases impose major economic consequences on Indian households through direct healthcare expenditures and productivity losses, particularly among economically vulnerable populations [14]. Their observations reinforce the present findings, which show that direct medical expenditure accounted for more than three-quarters of total diabetes-related costs.

This study contributes to the growing body of evidence indicating that healthcare utilization patterns significantly influence diabetes-related expenditures. Nearly all participants preferred allopathic care, with affordability and accessibility serving as the main determinants of healthcare utilization. Similar findings have been reported in studies from South Asia, where patients frequently bypass local facilities in favor of centers perceived to offer higher-quality diabetes care, leading to increased transportation costs and productivity losses. These results highlight the need to strengthen primary healthcare services and ensure the continuous availability of essential medicines to minimize avoidable out-of-pocket expenditures.

The findings have important implications for health policy. Although India has expanded financial protection through initiatives such as Ayushman Bharat and enhanced services under the National Programme for Prevention and Control of Noncommunicable Diseases, out-of-pocket expenditure remains a major source of healthcare financing for chronic diseases. Additionally, identifying households at highest risk of financial hardship should be integrated into diabetes management programs.

This study has several limitations. Its hospital-based cross-sectional design precludes causal inference and limits generalizability. Cost estimates were primarily based on participant recall and may therefore be subject to recall bias. In addition, intangible costs such as psychological distress, reduced quality of life, and presenteeism were not assessed and may have led to an underestimation of the overall societal burden.

## Conclusions

This study assessed the economic burden of diabetes using healthcare utilization and direct and indirect costs. T2DM imposes a significant household economic burden in rural North India, with direct costs comprising the largest share of expenditure. A substantial proportion of households allocated a large percentage of their monthly income to diabetes care, highlighting critical affordability challenges among socioeconomically vulnerable families. However, its hospital-based cross-sectional design limits generalizability, while participant recall may have introduced bias. Psychological distress, reduced quality of life, and presenteeism were not assessed. Policies that enhance access to affordable medicines, strengthen primary healthcare, expand financial protection, and reduce out-of-pocket expenditure are essential to mitigate the long-term economic consequences of diabetes and support progress toward Universal Health Coverage.

## Appendices

Appendix A 

**Table 6:** Study questionnaire

Section	Variable	Unit	Response options/measurement
Sociodemographic characteristics	Participant ID		
	Age	Years	
	Sex	Male/Female	
	Residence	Urban/Rural	
	Religion	Open response	
	Caste	General/OBC/SC/ST	
	Education	Illiterate/Primary/Secondary/Senior Secondary/Graduate	
	Occupation	Unemployed/Unskilled/Semi-skilled/Skilled/Retired	
	Marital status	Married/Unmarried/Widowed/Separated	
	Family type	Nuclear/Joint	
	Family size	Number of members	
	Family income	INR/month	
	Per capita income	INR/month	
	Socioeconomic status	Modified B.G. Prasad Classification	
Anthropometry	Height	cm	
	Weight	kg	
	Body mass index	kg/m²	
	Waist circumference	cm	
	Waist-hip ratio	Numeric	
Personal habits	Smoking	Yes/No; cigarettes/day; duration	
	Tobacco chewing	Yes/No	
	Alcohol consumption	Yes/No; frequency; quantity	
	Physical activity	Yes/No; duration (min/day); light/moderate/heavy	
Family history	Family history of diabetes	Mother/Father/Both/Cousin/None	
Clinical profile	Random blood sugar	mg/dL	
	Duration of diabetes	<5 years/5-10 years/>10 years	
	Physician visits (past 12 months)	Number	
	Hospitalizations (past 12 months)	Number	
	Diabetes-related complications	Nephropathy/Neuropathy/Retinopathy/Heart disease/Foot ulcer/None	
Symptoms (past week)	Dry mouth, increased thirst, decreased appetite, nausea/vomiting, abdominal pain, nocturia, morning headache, nightmares, night sweats, lightheadedness, shakiness/weakness, intense hunger, fainting, blurred vision, pain/numbness, skin rash/itching	Yes/No	
Dietary assessment	Type of diet	Vegetarian/Mixed	
	Meal frequency	1-5 meals/day	
	Meal timing	Fixed/Irregular	
	Type of salt	Iodized/Non-iodized	
	Type of cooking oil	Mustard/Refined/Vanaspati/Desi ghee	
	24-hour dietary recall	Breakfast, lunch, snacks, dinner, supper; calories and nutrient intake	
Health-seeking behavior	First healthcare provider consulted	Government hospital/Dispensary/Private doctor/AYUSH/Chemist/Home remedy	
	Delay in seeking treatment	Immediate/Delayed/Very delayed	
	Treatment system followed	Allopathy/Indian system/Naturopathy	
	Keeps medicines at home	Yes/No	
	Reason for choosing healthcare facility	Accessibility/Affordability/Availability/Family tradition	
	Perceived age-related illness	Yes/No	
	Distance to preferred health facility	<5 km/5-10 km/>10 km	
Cost category	Cost component	Unit of measurement	Amount (₹)
Direct medical costs	Consultation fee	Per visit/Monthly	
	Cost of medicines	Monthly	
	Laboratory investigations	Monthly	
	Hospitalization expenses	Per admission/Annual	
	Self-monitoring (glucometer, strips, and lancets)	Monthly	
	Treatment of diabetes-related complications	Annual	
Direct nonmedical costs	Transportation/travel	Monthly	
	Attendant/caregiver expenses	Monthly	
Indirect costs	Productivity loss (man-days lost)	Number of days × daily wage	
Total household cost	Total direct medical cost	Monthly/Annual	
	Total direct non-medical cost	Monthly/Annual	
	Total indirect cost	Monthly/Annual	
	Total household expenditure attributable to diabetes	Monthly/Annual	
